# LC3A-mediated autophagy elicits PERK-eIF2α-ATF4 axis activation and mitochondrial dysfunction: Exposing vulnerability in aggresome-positive cancer cells

**DOI:** 10.1016/j.jbc.2024.107398

**Published:** 2024-05-20

**Authors:** Nada Amer, Dina Hesham, Nouran Al-Shehaby, Hisham A. Elshoky, May Amer, Sameh Magdeldin, Manar Mansour, Khaled Abou-Aisha, Shahenda El-Naggar

**Affiliations:** 1 Tumor Biology Research Program, Basic Research Unit, Research Department, Children’s Cancer Hospital Egypt 57357, Cairo, Egypt; 2 Faculty of Pharmacy and Biotechnology, German University in Cairo (GUC), New Cairo, Egypt; 3 Proteomics and Metabolomics Research Program, Basic Research Unit, Research Department, Children’s Cancer Hospital Egypt 57357, Cairo, Egypt; 4 Department of Physiology, Faculty of Veterinary Medicine, Suez Canal University, Ismailia, Egypt

**Keywords:** aggresomes, autophagy, endoplasmic reticulum, inclusion bodies, MAP1LC3A, protein quality control, proteostasis, senescence

## Abstract

The unfolded protein response pathways (UPR), autophagy, and compartmentalization of misfolded proteins into inclusion bodies are critical components of the protein quality control network. Among inclusion bodies, aggresomes are particularly intriguing due to their association with cellular survival, drug resistance, and aggresive cancer behavior. Aggresomes are molecular condensates formed when collapsed vimentin cages encircle misfolded proteins before final removal by autophagy. Yet significant gaps persist in the mechanisms governing aggresome formation and elimination in cancer cells. Understanding these mechanisms is crucial, especially considering the involvement of LC3A, a member of the MAP1LC3 family, which plays a unique role in autophagy regulation and has been reported to be epigenetically silenced in many cancers. Herein, we utilized the tetracycline-inducible expression of LC3A to investigate its role in choroid plexus carcinoma cells, which inherently exhibit the presence of aggresomes. Live cell imaging was employed to demonstrate the effect of LC3A expression on aggresome-positive cells, while SILAC-based proteomics identified LC3A-induced protein and pathway alterations. Our findings demonstrated that extended expression of LC3A is associated with cellular senescence. However, the obstruction of lysosomal degradation in this context has a deleterious effect on cellular viability. In response to LC3A-induced autophagy, we observed significant alterations in mitochondrial morphology, reflected by mitochondrial dysfunction and increased ROS production. Furthermore, LC3A expression elicited the activation of the PERK-eIF2α-ATF4 axis of the UPR, underscoring a significant change in the protein quality control network. In conclusion, our results elucidate that LC3A-mediated autophagy alters the protein quality control network, exposing a vulnerability in aggresome-positive cancer cells.

The protein quality control (PQC) network is a complex system that regulates protein production, folding, and degradation (1, 2). Endoplasmic reticulum (ER)-resident chaperones are the initial checkpoint in the PQC network, whereby they assist newly synthesized proteins in folding into their correct three-dimensional structure (3, 4). Proteins that fail to fold correctly are either eliminated by the ubiquitin-proteasome system (UPS) or undergo autophagy-lysosome hydrolysis (2, 3). The unfolded protein response (UPR) is another crucial mechanism that cells use to maintain ER protein homeostasis, with three stress sensors: Protein Kinase R-like ER Kinase (PERK), Activating Transcription Factor (ATF6), and Inositol Requiring Element 1 (IRE1), regulating protein trafficking, folding, and degradation (4). The UPR is activated in response to protein misfolding and helps cells cope with this stress by increasing the production of chaperones, halting protein synthesis, and promoting the degradation of misfolded proteins (5). In recent years, protein compartmentalization into specific cellular sites, forming membrane-less bimolecular condensates, was found to be a critical component of the PQC network (6, 7). Among different types of condensates, the aggresome has emerged as a specialized structure formed by the collapse of the intermediate filament vimentin at the microtubule organizing center (MTOC) (7, 8, 9). Ensuing experiments further supported the role of aggresome formation as a protective mechanism against the accumulation of misfolded proteins before final removal by autophagy (10, 11, 12).

Autophagy is a catabolic process that occurs at a basal level or is induced by stress, such as nutrient deprivation (13). Autophagy is classified into three subtypes: macroautophagy, microautophagy, and chaperone-mediated autophagy, based on the cargo delivery route to the lysosome (2). When macroautophagy is initiated (hereafter referred to as autophagy), an isolation membrane expands around the cargo and closes to form autophagosomes, then fuse with the lysosome for final degradation (13). Selective forms of autophagy were also identified where specific cellular compartments or cargo were eliminated (14, 15, 16). The evolutionary conserved autophagy-related (ATG) proteins are an integral autophagy component (17, 18). In mammalian cells, the yeast Atg8 orthologs are classified into two families: microtubule-associated protein 1A/1B light chain (MAP1LC3, referred to as LC3), consisting of LC3A, LC3B, and LC3C, and GABA type A receptor-associated protein (GABARAP), consisting of GABARAP, GABARAPL1, and GABARAPL2 (19). The LC3 paralogs are ubiquitin-like proteins that play an essential role in cargo recognition, engulfment, and vesicle closure (18). LC3B is the most extensively studied mammalian LC3 paralog and is widely used for assessing autophagy flux (20). Recently, differences between LC3 paralogs pertaining to their localization, regulation, molecular function, and interactome have been recognized (20, 21, 22). This is further supported by the observation that LC3 members interact with specific adapters for cargo recruitment, thereby providing functional specialization in cargo selection (23). Studies have shown that LC3A exhibits distinct expression patterns and functions from LC3B and LC3C. Furthermore, gene mutation and epigenetic silencing by promoter methylation have been identified in various human cancers, including multiple myeloma, breast, colon, and lung (24, 25, 26). Silencing of LC3A has been linked to impaired autophagy flux, increased cellular invasion, and a poor disease prognosis. This suggests that LC3A may have a differential and specific role in regulating protein homeostasis and serving as a tumor suppressor (27, 28, 29). These findings have prompted the investigation of the role of LC3A-mediated autophagy in maintaining cellular proteostasis.

In the current study, we investigate the role of LC3A independent of LC3B in cancer cells that inherently exhibit the presence of aggresomes. Our findings indicate that the activation of LC3A is associated with cellular stress response governed by the PERK-eIF2α-ATF4 axis of the UPR pathway, resulting in altered mitochondrial dynamics and stress-induced senescence. Additionally, inhibiting LC3A-mediated autophagy in aggresome-positive cancer cells has detrimental effects on cell viability. These results support the significance of specialized autophagy in cellular homeostasis and provide valuable biological insight into how cancer cells exploit PQC for survival. This warrants further investigation, as it could empower the development of targeted therapeutic strategies in diseases with known proteopathy associations.

## Results

### LC3A activation and lysosomal blockage disrupt cellular homeostasis, while sustained LC3A activation induces cellular senescence

**Figure 1:**
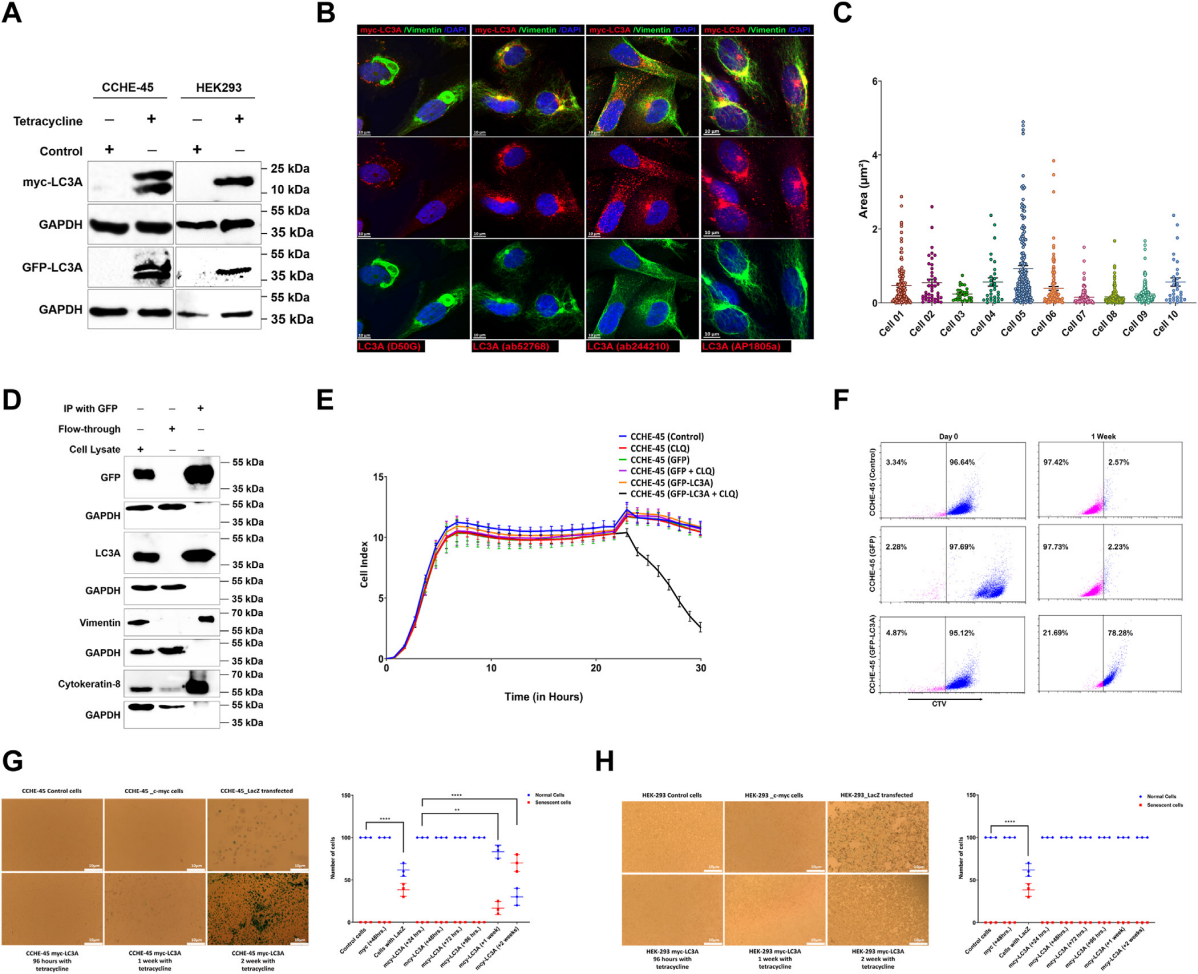
**Activation of LC3A-mediated autophagy in aggresomes positive cells.***A*, Western blot analysis of CCHE-45 and HEK-293 cell lysates for LC3A expression post-induction with tetracycline. LC3A expression was detected using an anti-LC3A or anti-GFP antibody in myc-LC3A and GFP-LC3A transfected cells, respectively. GAPDH served as a loading control. *B*, double immunofluorescence staining for LC3A (*red*) and vimentin (*green*) to assess antibody specificity in myc-LC3A-induced cells. DAPI was used for nuclear staining. Representative images are shown from three independent experiments. *C*, scatter plot of the number and sizes of GFP-LC3A-positive puncta in 10 cells using ZEN blue segmentation analysis. *D*, immunoprecipitation analysis using anti-GFP antibody following induction of GFP-LC3A expression for 48 h. Samples were then analyzed by immunoblotting using antibodies against GFP, LC3A, vimentin, and CK8. Whole-cell lysates were used as a positive control, while the residual lysate was used as a negative control. *E*, impedance monitoring was performed for the following conditions; CCHE-45 untransfected cells, CCHE-45 cells stably transfected with GFP-LC3A, GFP-LC3A with CLQ, GFP, and GFP with CLQ. The X-axis represents time in hours. The Y-axis represents the cell index obtained from the RTCA software.Three biological replicates were performed and the data is presented as mean ± SD. *F*, representative flow cytometric analysis of CellTrace Violet staining showing CCHE-45 cell proliferation at day 0 and after 1 week, with and without expression of GFP-LC3A. *G* and *H*, senescence-associated B-galactosidase (SA-B-Gal) was assessed in CCHE-45 and HEK293 cells following induction of myc-tag or myc-LC3A expression using tetracycline. Cells were monitored for 2 weeks, and the number of SA-B-Gal-positive cells was counted and averaged from three independent experiments. The reported values represent the mean ± SD. The data represent three independent biological replicates and the statistical significance was determined using two-way ANOVA analysis to compare different conditions, with ∗∗*p* < 0.01 and ∗∗∗∗*p* < 0.0001 denoting significant differences.

In this study, we aim to characterize the role of LC3A-mediated autophagy in choroid plexus carcinoma cells (CPC) that inherently harbor aggresomes. Aggresomes are well-known sites where misfolded proteins are sequestered, ultimately leading to their degradation through autophagy. In a previous study, we showed *LC3A* silencing in CCHE-45 cells, which was attributed to intergenic CpG island methylation (29). The inactivation of LC3A expression has been reported in various tumors, including lung, breast, and colon cancers (24, 30). Notably, this inactivation is associated with aggresome formation, specifically in multiple myeloma (24, 31). Hence, our primary objective is to explore the consequences of activating LC3A on the dynamics of aggresomes, and its subsequent impact on overall cellular homeostasis. We generated tetracycline-inducible myc-LC3A and GFP-LC3A fusion proteins by cloning *LC3Av1* cDNA downstream of myc or GFP tags (Fig. S1*A*). Plasmids were stably transfected in CCHE-45 (LC3A- negative, aggresomes-positive) and HEK293 (LC3A-negative, aggresomes-negative) cells (32, 33). We verified the mRNA and protein expression levels of LC3A in both systems, respectively (Figs. S1*B* and 1*A*). Consistent with our previous report, tetracycline-induced myc-LC3A expression showed puncta distribution throughout the cells without serum starvation, as observed by immunofluorescence using different anti-LC3A antibodies (Figs. 1*B* and S1*C*). Similarly, GFP-LC3A puncta were detected as early as 24 h post-induction (Fig. S1*D* and Movie 1), with approximately 97.2% of puncta colocalizing with lysosomes at 48 h (Manders' coefficient factor =0.9723) (Fig. S1*D* and Table S1). In GFP-LC3A CCHE-45 cells (N = 10), the number of puncta ranged from 32 to 156, and their average area varied between 0.04 and 4.89 μm^2^. (Fig. 1*C* and Table S1). The variation in average puncta numbers and area reflects the dynamic nature of autophagy activation, suggesting that different cells within the population exhibit varying degrees of autophagosome formation. Additionally, a GFP-LC3A signal was detected in the nucleus after more than 50 h of induction (Movies 1 and 2). In contrast, the expression pattern of GFP-LC3A in HEK293 cells exhibited a diffused distribution with no discernible puncta (Fig. S1*E*). Concurrently, vimentin in HEK293 cells exhibited well-defined filaments (Fig. S1*F*). However, upon treatment with MG132, a proteasome inhibitor, the control and GFP-LC3A-expressing HEK293 cells displayed a perinuclear ring-shaped collapsed vimentin. Additionally, GFP-LC3A transitioned from a diffused pattern to small puncta scattered around the newly formed aggresomes (Fig. S1*F*). To further elucidate the relationship between aggresomes and LC3A, independent of LC3B, and to investigate whether the simultaneous activation of LC3A in CCHE-45 cells is cell-line-specific or a general stress response triggered by aggresomes, we subjected SH-SY5Y neuroblastoma cells to serum starvation. After 5 h of serum starvation in Hank's Buffered Salt Solution, LC3B-positive autophagic puncta were detected and further intensified with the addition of chloroquine (CLQ), supporting the induction of LC3B-mediated autophagy, with no change observed in the LC3A pattern (Fig. S1*G*). However, the induced formation of aggresomes in SH-SY5Y cells after MG132 treatment was accompanied by a transition in LC3A expression from a diffuse to a distributed cytoplasmic punctate pattern, supporting the induction of LC3A-mediated autophagy (Fig. S1*H*). These observations, involving both exogenous LC3A in HEK293 and endogenous LC3A in SH-SY5Y after proteasome inhibition, provide valuable insights into the role of LC3A in the PQC network of cells. Moreover, they establish a correlation between LC3A and aggresomes, revealing distinct cellular triggers for LC3A and LC3B. To confirm whether puncta clustering at the aggresomes indicated a physical interaction between LC3A and aggresomes in CCHE-45 cells, we performed immunoprecipitation using an anti-GFP antibody on GFP-LC3A-induced cells after 48 h. The precipitated samples revealed the presence of vimentin and cytokeratin-8 intermediate filaments, components of the aggresome cage in CCHE-45 cells, indicating the physical interaction of LC3A and the aggresome cage (Fig. 1*D*). To monitor LC3A-mediated autophagy flux, LC3A expression was induced, and cells were treated with the lysosomal inhibitor CLQ. Interestingly, a decline in cell viability was observed shortly after treatment, which was not observed when cells were treated with CLQ alone, serum-starved, or serum-starved plus CLQ treatment (Fig. 1*E* and Movie 3). Due to the observed cell death resulting from LC3A induction and CLQ treatment, our hypothesis was that LC3A activation alone is not detrimental to cell survival and CCHE-45 cells might display an elevated reliance on the autophagy-lysosome pathway, making them more susceptible to inhibition of lysosomal degradation. However, sustained expression of LC3A may result in cell death. To explore this further, we extended our cell monitoring for 100 h following the induction of LC3A expression. We detected no significant alterations in the cell index (Fig. S1*E*). This indicated that LC3A expression alone is insufficient to trigger cell death. Nevertheless, after 96 h, we observed changes in the cells' physical characteristics and nuclei. Consequently, we tracked cell proliferation and monitored cell division by assessing CellTrace Violet's (CTV) dilution using flow cytometry. The proliferation of CCHE-45 cells remained unchanged in both control and those expressing GFP, whether in the presence or absence of tetracycline (Fig. 1*F*). We monitored cell proliferation for up to 1 week in culture. However, we observed a substantial 80% decline in cell proliferation during the prolonged expression of GFP-LC3A for 1 week, which persisted over the subsequent week (Fig. 1*F* and Table S1). Importantly, we avoided relying on monitoring cell proliferation beyond 7 days to ensure that our data would not be affected by the potential deterioration effects of the CTV dye. These findings collectively indicate that the prolonged expression of LC3A can suppress the proliferation of aggresome-positive cells. Based on our collective previous observations, we investigated whether the cells were entering a state of senescence. Our findings revealed that the sustained expression of LC3A for at least 1 week induced senescence in CCHE-45 cells (Fig. 1*G*), whereas it did not affect aggresome negative HEK293 cells (Fig. 1*H*).

### Expression of LC3A elicits global cellular stress altering the proteostasis network

**Figure 2:**
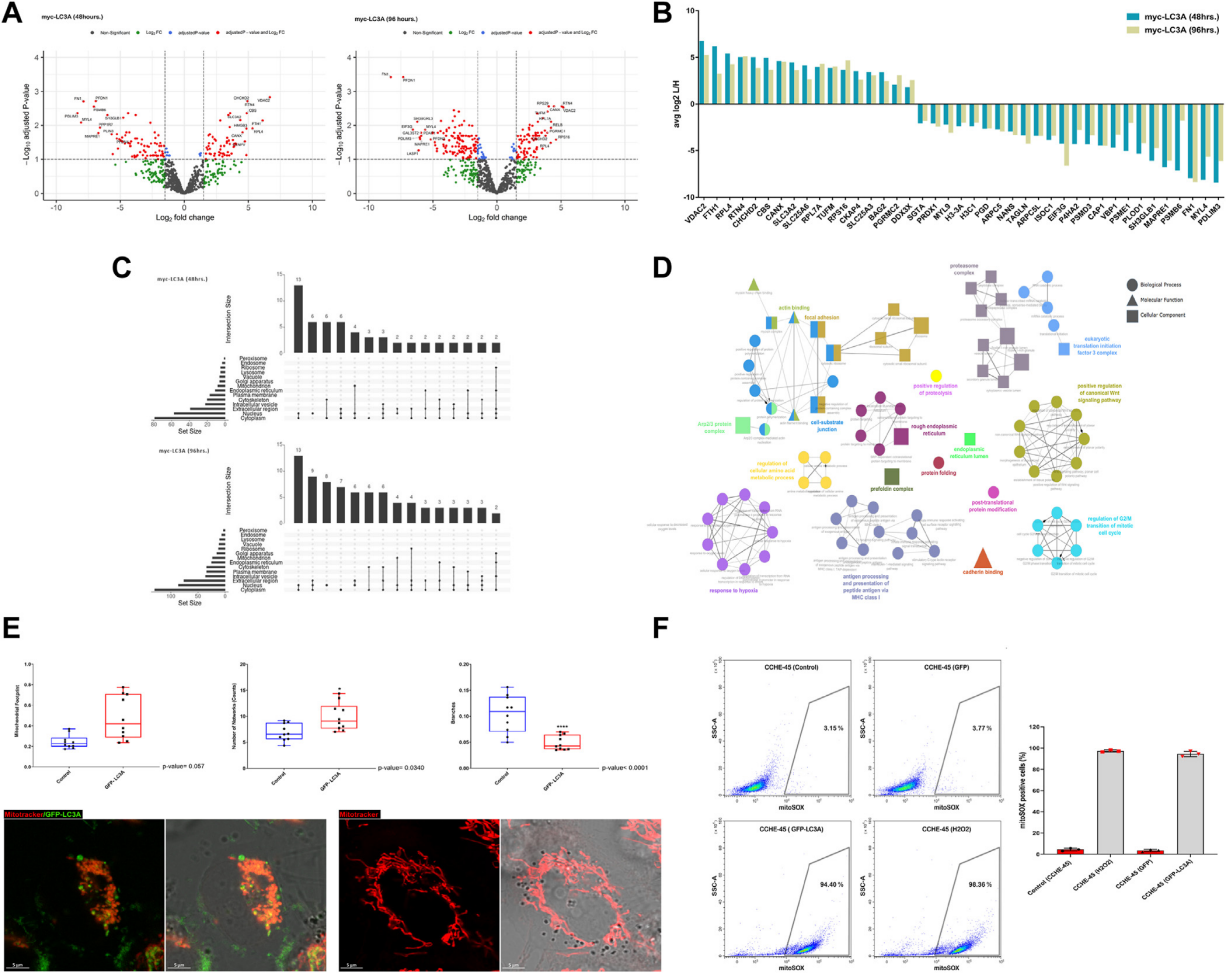
**The proteome landscape associated with the expression of LC3A protein.***A*, volcano plot of differentially expressed proteins after the induction of LC3A expression for 48 and 96 h. The log2 FC on the X-axis and log10 adjusted *p*-value on the Y-axis. The *horizontal line* represents the cutoff of the adjusted (*p*-value < 0.05), and the vertical lines represent the cutoff of the log2 FC (1 and −1). *B*, bar plot of the average log2 fold change of the Light/Heavy ratio identified from SILAC for the 41 common proteins. The *blue* and *green bars* represent protein FC at 48 and 96 h, respectively. *C*, NUpSet plot for DEPs subcellular localization using SubcellularRVis. Each set's total number of proteins is represented as a bar chart. Each row corresponds to a cellular compartment where filled-in cells show the compartment and the intersection with the other compartment. *D*, network visualization of GO for biological process, molecular function, and cellular component for differentially expressed proteins shared at 48 and 96 h following induction of LC3A expression. The nodes in the network represent the GO terms, while the edges connecting them represent the relationships between the terms. *E*, confocal microscopy images using a 60× objective lens of mitochondria labeled with MitoTracker in CCHE-45 cells after 48 h of induction of GFP-LC3A expression. Barplots represent counts obtained from the MINA analysis across three different experiments with the reported values representing the mean ± SD of independent biological replicates, statistical significance was determined using one-way ANOVA analysis, with ∗*p* < 0.05 and ∗∗∗∗*p* < 0.001 denoting significant differences. *F*, flow cytometry analysis for mitoSOX in CCHE-45 control cells, cells expressing GFP, and cells expressing GFP-LC3A. H2O2 was utilized as a positive control for ROS stress. The bar plot represents the percentages of mitoSOX-positive cells across three different replicates, with the reported values representing the mean ± SD.

The presence of LC3A-positive puncta in the absence of serum starvation and the subsequent dissolution of the vimentin cage surrounding aggresomes strongly imply that LC3A-mediated autophagy plays a crucial role in identifying and removing materials contributing to aggresome formation. Additionally, a senescence phenotype indicates that prolonged LC3A-mediated autophagy is linked to the accumulation of cellular stress signals over time. Hence, we employed quantitative proteomics to identify the protein network changes associated with LC3A expression. We performed quantitative proteomics using SILAC for the myc-LC3A stable clone 48 and 96 h post-induction with tetracycline to capture early and late events. CCHE-45 cells expressing myc-tag were used as controls for the effect of myc-tag and tetracycline. We identified 808 and 872 proteins, in three independent biological replicates at 48 and 96 h, after excluding common proteins with myc control cells (Table S2). Correlation scores were calculated between the different biological replicates based on the abundance of identified proteins in each sample (Fig. S2, *A* and *B*). A total of 88 and 145 were differentially expressed proteins (DEPs) at 48 h and 96 h, respectively (Fig. 2*A*). Among DEPs, 41 proteins were shared between the two time points (Fig. S2*C*). Shared DEPs displayed similar expression patterns, while the fold change was generally higher at 48 h, with 24 proteins downregulated, and the other 17 upregulated (Figs. 2*B* and S2*D*).

Subcellular localization prediction was then used to gain further insight into the spatial distribution of the DEPs. Most DEPs at 48 h were localized to the nucleus, cytoplasm, and ribosomes. However, at 96 h, proteins were mainly localized to the ER, mitochondria, Golgi, and intracellular vesicles (Fig. 2*C* and Table S3). Enrichment analysis of the biological processes for the exclusive early event (48 h) identified regulation of gene silencing, chromatin condensation, and conformation change in DNA (Fig. S2*E* and Table S4). After 48 h of LC3A induction, the protein interactome was associated with chromatin condensation, tri-H3, H4 methylation of histone, and epigenetic maintenance. Recently, a link between chromatin remodeling and autophagy following rapamycin treatment was found to be mediated by the non-canonical eukaryotic initiation factor 3 (eIF3) (34, 35). A similar mechanism may occur since the down-regulated DEPs are two members of the eIF3 complex; eIF3C and eIF3G. On the other hand, gene ontology (GO) analysis of the biological process for the exclusive late events (96 h) was significantly enriched in the cellular response to unfolded protein, protein-targeting to the ER, cellular response to osmotic stress, negative regulation of mitochondria, ER to Golgi vesicles transport and antigen processing and presentation (Fig. S2*F* and Table S4). Common DEPs identified a broad range of proteostasis pathways enrichment, including protein assembly, protein translation initiation, protein folding, targeting to the ER, positive regulation of proteolysis, and RNA catabolic process (Fig. 2*D* and Table S4).

Interestingly, the top-upregulated protein in both time points was the outer mitochondrial membrane protein voltage-dependent anion channel 2 (VDAC2) (36). The VDAC family of proteins is essential in several mitochondrial functions, including metabolite exchange, calcium transport, and apoptosis (36, 37). Recent evidence, however, supports the role of VDAC2, specifically in mitochondrial Ca^2+^ influx through contact sites between mitochondria and ER (38). Furthermore, the increase in the protein FKBP1A, a modulator of calcium channels, observed at 96-h, along with the enrichment in the pathway associated with ER ryanodine-sensitive calcium release channels (RyR2), provides strong support for potential changes in intracellular calcium levels. These changes could potentially be caused by ER calcium leak and activation of the UPR, possibly triggering a compensatory mechanism involving mitochondria. Consequently, we investigated the role of mitochondria as a mediator of ER-induced stress and cellular allostasis. To determine alterations in the mitochondria environment following LC3A expression induction, we used changes in mitochondrial morphology as a readout. Notably, CCHE-45 cells expressing GFP-LC3A exhibited a significant reduction in mitochondrial branching and increased interconnected networks, indicative of a more compact and circular mitochondrial morphology. Conversely, we observed a general increase in mitochondrial footprint, although these changes were not associated with large-scale disruption of mitochondria content since there was no significant change (Fig. 2*E*). These results suggest that in response to LC3A-induced autophagy, cells navigate ER stress by mitochondria-mediated mechanism, possibly by buffering cytoplasmic Ca^2+^ levels. To assess the impact of the morphological changes in mitochondria on their function, we employed the MitoSOX dye to evaluate mitochondrial function *via* flow cytometry under different conditions. CCHE-45 cells stably expressing GFP-LC3A displayed an increase in the intensity of the MitoSOX dye. This increase was comparable to the positive control H_2_O_2_. However, no similar pattern was observed in GFP-expressing CCHE-45 cells (Fig. 2*F*). The heightened intensity of the MitoSOX fluorescence signal indicates elevated levels of mitochondrial superoxide, suggesting an association with oxidative stress. This finding underscores the potential link between LC3A-induced autophagy, alterations in mitochondrial morphology, and increased mitochondrial superoxide levels.

### LC3A-mediated autophagy is associated with the activation of the PERK-eIF2α-ATF4 axis of the UPR

**Figure 3:**
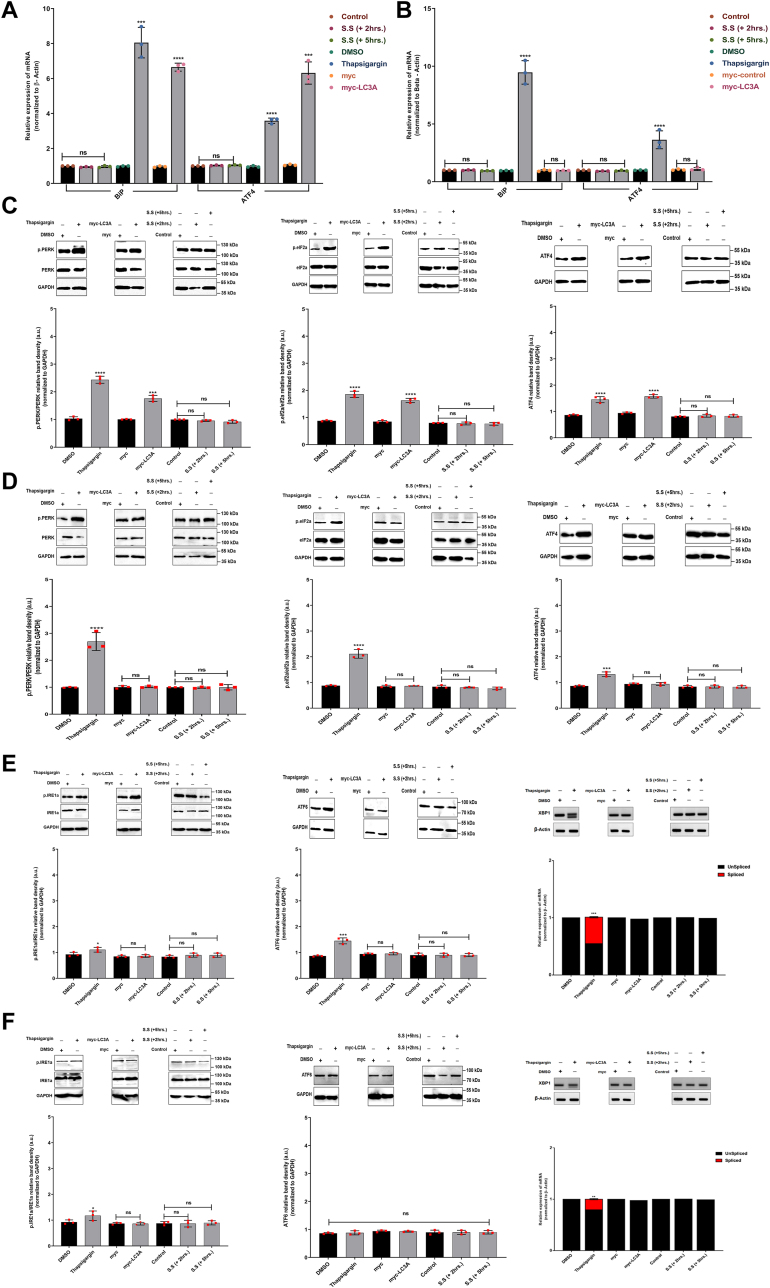
**UPR analysis following induction of LC3A expression.***A* and *B*, real-time PCR analysis depicting the mRNA expression levels of Bip and ATF4 under various experimental conditions normalized to the β-actin as a housekeeping gene for CCHE-45 and HEK293 cells, respectively. A serum-starved group was labeled as “S.S”. The reported values representing the mean ± SD of three independent biological replicates, statistical significance was determined using one-way ANOVA analysis, with ns= Non-significant, ∗∗*p* < 0.01, ∗∗∗*p* < 0.001 and ∗∗∗∗*p* < 0.0001 denoting significant differences. *C* and *D*, Western blot analysis for PERK pathway activation in CCHE-45 and HEK293 cells, respectively, expressing myc or myc-LC3A for 48 h. *E* and *F*, Western blot analysis for IRE1 and ATF pathway activation in CCHE-45 and HEK293 cells, respectively. XBP1 reverse transcriptase analysis was performed for the detection of XBP1 splicing. Quantification was performed using XBP1 expression relative to the β-actin gene. In all experiments, cells were treated with Tg for 6 h. Western blot analysis was quantified using the relative density of the desired protein compared to the loading control, GAPDH. Plots depict mean ± SD of three independent biological experiments. Statistical significance was determined using one-way ANOVA analysis, with ns= Non-significant, ∗*p* < 0.05, ∗∗*p* < 0.01, ∗∗∗*p* < 0.001 and ∗∗∗∗*p* < 0.0001 denoting significant differences.

Given the profile of DEPs associated with ER, mitochondria resident proteins, trafficking between the ER and Golgi, and alterations in mitochondria morphology, we hypothesized that LC3A-mediated autophagy in our cellular context correlates with the ER stress response activation. Moreover, since phosphorylation events regulate the UPR pathways, we examined its activation despite the absence of its members in the proteomics data. Accordingly, we investigated the three UPR stress sensors, PERK, ATF6, and IRE1-α, in both CCHE-45 and HEK293 cells after inducing the expression of LC3A for 48 h. We used thapsigargin (Tg) as a positive control to assess ER stress activation and treated the cells for 6 h. Furthermore, serum starvation for 2 and 5 h was used to assess whether LC3B-mediated autophagy would elicit similar effects. We observed a significant increase in the expression levels of the ER lumenal chaperone Bip in both Tg-treated and myc-LC3A-induced CCHE-45 cells (Fig. 3*A*). Similarly, an increase in the expression of ATF4, a downstream transcription factor of the PERK arm, was detected in Tg-treated and myc-LC3A-induced cells (Figs. 3*A* and S3). On the other hand, only Tg-treated HEK293 cells had an increase in Bip and ATF4 expression levels (Figs. 3*B* and S4). The increased expression of ATF4 was associated with the phosphorylation of PERK and eIF2α (Figs. 3*C* and S3). Furthermore, the activation of the PERK arm was only associated with LC3A-mediated autophagy since serum starvation did not induce the same response (Fig. 3, *A* and *C*). Further confirming that such effect is specific to LC3A and not LC3B mediated autophagy. In contrast, we did not detect activation of the PERK arm in HEK293 cells following LC3A activation (Fig. 3*D*). Only Tg treatment activated the PERK pathway in HEK293T cells (Fig. 3*D*). Hence confirming that the activation of the PERK arm is LC3A-mediated only in aggresome-positive cells. Next, we examined XBP1 splicing, a downstream marker of IRE1α activation, and ATF6. Our findings show that we could only detect XBP1 splicing in Tg-treated CCHE-45 and HEK293 cells, whereas ATF6 exhibited increased expression rather than splicing (Fig. 3, *E* and *F*).

## Discussion

Based on these findings, we propose that CCHE-45 cells employ a strategy of sequestering misfolded or aggregated proteins and dealing with protein overload by forming aggresomes. Upon the introduction of LC3A, these cells trigger LC3A-mediated autophagy, directing the cargo from aggresomes toward the autophagosomal-lysosomal system for degradation. Once cellular commitment to LC3A-mediated autophagy is established, the process becomes reliant on LC3A for maintaining proteostasis, and any interference with lysosomal degradation proves deleterious to the cellular milieu. This commitment also involves the ER and mitochondria's active engagement in managing cellular stress. While these mechanisms can effectively mitigate stress stemming from protein misfolding, their prolonged activation may exert detrimental effects on cancer cells.

In response to LC3A-induced autophagy, we observed significant alterations in the mitochondrial morphology of CCHE-45 cells, characterized by a reduction in branching and an increase in interconnected networks, indicative of a more compact and circular mitochondrial structure (39). Although these changes did not disrupt overall mitochondrial content, as evidenced by a consistent footprint, the morphological shifts suggested a mitochondria-mediated response to ER stress. A substantial increase in mitochondrial superoxide levels in CCHE-45 cells expressing GFP-LC3A, underscores a connection between LC3A-induced autophagy, mitochondrial morphology alterations, and oxidative stress. These findings align with proteomics analysis, unveiling a significant enrichment in the 'response to hypoxia' biological process in LC3A-expressing. This suggests that mitochondrial dysfunction contributes to the cellular response to LC3A expression, emphasizing the intricate interplay between autophagy and mitochondrial dynamics.

The UPR constitutes an adaptive cellular mechanism for sensing and responding to stress. When initiated, it can serve a dual role: it can alleviate stress by reducing protein synthesis and expanding the ER capacity to restore cellular homeostasis. Alternatively, it can activate processes leading to cell death when unresolved stress conditions remain (40). Activation of the UPR involves three pathways: PERK, ATF6, and IRE1. Their activation is triggered by an increase in the ER chaperone Bip protein and its subsequent release from the luminal domains of these proteins (41). In our study, the expression of LC3A was exclusively associated with the activation of the PERK pathway. Recent evidence points to a paradoxical regulation of IRE1 under sustained ER stress conditions, where PERK can inhibit the adaptive responses mediated by IRE1 (40). These findings offer a plausible explanation for the absence of IRE1 activation in our experimental model despite the increased expression of Bip. The lack of ATF6 pathway activation in CCHE-45 cells may be attributed to the upregulation of VDAC2, which is known to suppress the functioning of the ATF6 branch within the UPR (42). Consequently, we can propose that LC3A-mediated autophagy in cells with aggresome accumulation activates the PERK pathway to resolve ER stress. However, the absence of IRE1 and ATF6 activation implies a potential predisposition towards cell death which explains the detrimental effects of CLQ treatment following LC3A expression.

In conclusion, our study demonstrates that LC3A orchestrates basal autophagy and effectively resolves aggresome formation. Notably, inhibiting lysosomal degradation in the presence of LC3A elicits deleterious effects on cellular homeostasis, warranting exploration as a prospective therapeutic avenue. Additionally, the sustained activation of LC3A is linked to ER stress, initially mitigated through mitochondrial mechanisms but culminating in cellular senescence. While our investigation did not encompass measurements of Ca^2+^ dynamics, our findings strongly suggest a potential involvement of Ca^2+^ in mediating the intricate interplay between the ER, mitochondria, and the induction of senescence, thereby emphasizing the need for dedicated future investigations.

## Experimental procedures

### Plasmids and cloning

The pcDNA4/TO/myc-His A (Invitrogen, K1030-02) was used to generate; myc-LC3Av1, GFP, and GFP-LC3Av1 expression vectors using XhoI and AgeI restriction enzymes. Gene synthesis and cloning were performed by Eurofins Scientific Company.

### Cell culture, generation of inducible T-Rex stable clone, and drug treatment

CCHE-45 and SH-SY5Y cells were cultured in Roswell Park Memorial Institute-1640 (RPMI 1640) medium (Gibco, 52400025), while HEK293 cells were cultured in Dulbecco's modified Eagle's (DMEM) medium (Gibco, 41966029). Both media were supplemented with 10% fetal bovine serum (FBS) (Gibco, 10270106) and 1% penicillin-streptomycin (Gibco, 15140122). The cells were maintained under standard cell culture conditions at 37 °C and a CO_2_ concentration of 5%. Both cell lines were verified to be free from *Mycoplasma* contamination. For the generation of stable clones, cells were first cultured in their respective medium supplemented with 10% tetracycline-reduced FBS (Thermo Scientific, A4736301) for 24 h before transfection. The transfection process involved cotransfecting the cells with a mixture of the gene of interest construct and the pcDNA6/TR regulatory vector (Invitrogen, K1030-02) at a ratio of 1:6 using Lipofectamine 3000 transfection reagent (Invitrogen, L3000015) according to the manufacturer's instructions. However, the cells were incubated with the DNA-lipid complex for 10 to 15 min before adding the complete culture medium. Twenty-four hours after transfection, cells were washed, and fresh medium supplemented with 10% tetracycline-reduced FBS was added. After an additional 48 h, transfected cells were maintained in a selective medium containing 10% tetracycline-reduced FBS, 5 μg/ml and 250 μg/ml for CCHE-45, and 3 μg/ml blasticidin and 125 μg/ml zeocin for HEK293. Cells were maintained in the selective medium for 4 weeks until distinct focal points (foci) developed. Twenty different foci were selected and expanded to screen for the expression of LC3A. To induce LC3A expression, tetracycline was added to the cells to a final concentration of 1 μg/ml, and the cells were incubated for at least 24 h at 37 °C. For the induction of LC3B-mediated autophagy, cells were serum-starved in Hank’s balanced salt solution (Lonza) for 2 and 5 h. To evaluate autophagy flux, CLQ (Enzo Life Sciences) was added to the cell culture medium at a final concentration of 50 μM to block the fusion between autophagosomes and lysosomes. The Tg drug, which inhibits the ER Ca^2+-^ATPase, was used as a positive control to induce ER stress (1, 43). Tg was added to the cells’ culture medium at a final concentration of 1 μM for 6 h. The cells were harvested for further analysis at the indicated time point. Hydrogen peroxide (H_2_O_2_, 30% w/v) (Adwic, H0038111) was utilized as a positive control to induce reactive oxygen species (ROS) stress in CCHE-45 and HEK 293 cells. H_2_O_2_ was added to the culture media at a final concentration of 20 μM for 4 h after treatment. Cells were harvested for subsequent analyses. 5 μM of the proteasome inhibitor MG132 (Cell Signaling, 2194S) was added to cells for 6 h to induce the formation of aggresomes in HEK293 and SH-SY5Y cells.

### RT-PCR and real-time PCR

Total RNA was extracted using TRIzol reagent (Invitrogen, 15596-026), and the resulting RNA was reverse transcribed using the RevertAid First Strand cDNA Synthesis Kit (Thermo Scientific, K1622). Real-time PCR was conducted on the CFX96 Touch Real-Time PCR Detection System (Bio-Rad) using Maxima SYBR Green qPCR Master Mix (2×) (Thermo Scientific, K0251). Quantification analysis was performed using the comparative threshold cycle (Ct) method, with the Ct values of each gene normalized to the Ct value of ß-actin. All experiments were performed in triplicate. The fold change in gene expression was determined using the equation 2^-ΔΔCt^ (44). To detect XBP1 gene splicing, PCR was conducted with DreamTaq Green PCR Master Mix (2×) (Thermo Scientific, K1081). All protocols were performed according to the manufacturer's instructions with the primers described in (Table S5).

### SDS-PAGE and Western blot analysis

For protein analysis, cells were lysed using a lysis buffer containing 8M urea and 500 mM Tris-HCl (pH 8.5), and a protease inhibitor cocktail (Thermo Scientific, A32955). The total protein concentration was determined using a bicinchoninic acid (BCA) protein assay kit (Thermo Scientific, 23225). Equal amounts of proteins were resolved by 12% SDS-PAGE at 100 V for 2 h and transferred to PVDF membranes (Thermo Scientific, 88518 and 88520). The membranes were then blocked with 5% w/v nonfat dry milk for 1 h at room temperature. Subsequently, membranes were probed with specific primary antibodies (listed in Table S6) overnight at 4 °C. After three washes, the membranes were incubated with secondary antibodies for 1 h at room temperature. Detection was performed with Pierce ECL plus W.B. substrate (Thermo Scientific, 32132) and scanned using the ChemiDoc MP Imaging System (Bio-Rad) with all bands in the linear range of detection. ImageJ was utilized for quantitative analysis of protein levels and statistical comparisons among different treatments.

### Immunofluorescence and confocal microscopy

For immunofluorescence staining, cells were seeded in 6-well plates on microscope cover glasses (22 mm × 22 mm) coated with Poly-D-Lysine (Gibco, A3890401). After treatment, cells were fixed with 4% paraformaldehyde (Sigma-Aldrich, P6148) in PBS for 15 min and permeabilized using 0.3% Triton X-100 (Sigma-Aldrich, 93420) in PBS for 15 min. Blocking buffer (0.3% Triton X-100 and 5% FBS in PBS) was added to cells for 1 h at room temperature. Fixed cells were incubated overnight at 4 °C with primary antibodies in the antibody-dilution buffer (0.3% Triton-X and 1% BSA in PBS) in a wet chamber. Detailed information about the antibodies used is listed in (Table S2). After three washes with PBS, the cells were incubated with Alexa Fluor-conjugated secondary antibodies for 1 h in the dark at room temperature, followed by incubation with DAPI (Invitrogen, D1306) for nuclear staining. Positive-charged slides were then mounted using Prolong Gold (Invitrogen, P36930). LysoTracker Deep Red dye (Invitrogen, L12491) was used to label lysosomes at a 1:10,000 dilutions. For visualization of the mitochondria, CCHE-45 stable clones were grown on live imaging cell culture plates, and GFP-LC3A expression was induced with tetracycline for 24 h before adding Mitotracker Red CMXRos (Invitrogen) (1:1000 dilution) for 30 min at 37 °C. The cells were washed with PBS, and a fresh medium was added. Live imaging was performed using a ZEISS LSM 980 confocal microscope equipped with an Airyscan 2 detector confocal microscope at 60× magnification with a Plan-Apochromat 60×/1.4 Oil DIC (UV) VIS-IR M27 objective lens 24 h after the induction. The cells were maintained at 37 °C with 5% CO_2_ during imaging for 24 h.

### Mitochondrial reactive oxygen species (ROS) measurement

CCHE-45 cells were treated with 5 μM MitoSOX Red mitochondrial superoxide indicator (Invitrogen, M36007) for 30 min at 37 °C in the dark, following the manufacturer’s instructions. The unstained cells served as the control for each sample. Approximately 40,000 gated events were acquired for each sample on a CytoFLEX (Beckman Coulter) and analyzed using CytExpert software. Dead cells and debris were excluded based on forward scatter and side scatter measurements. All analyses were gated on control of unstained CCHE-45 cells, determined by morphologic identification (forward scatter *versus* side scatter). The mean value ± standard deviation (SD) for the percentage of Mitosox-positive cells was calculated.

### Image analysis

Fiji (ImageJ) software was used for image analysis. The MitoTracker channel threshold was set using ImageJ's Auto Threshold plugin. Mitochondrial network parameters, length, and branching were then quantified using the Mitochondrial Network Analysis (MINA) plugin in ImageJ (45). Statistical analysis was performed using GraphPad Prism software version 8. The segmentation analysis module in ZEN 3.3 software (Blue edition) was employed to identify and count the puncta representing positive autophagosomes. Parameters such as thresholds and size criteria were adjusted to ensure accurate detection and quantification of the puncta. The software automatically detected and counted the puncta based on the predefined segmentation criteria. To ensure accuracy, the results of the automated puncta counting were manually validated and refined if necessary. The counted puncta were recorded and subjected to further statistical analysis or comparisons. Linear unmixing analysis was performed using ZEN 3.3 software. Channels corresponding to different labels were selected, and the linear unmixing algorithm was applied to separate the contribution of each label from the mixed signal in the acquired images. This process facilitated the separation of specific signals corresponding to individual colors, reducing spectral overlap and enabling enhanced visualization and analysis of the target structures. Subsequently, quantitative measurements, such as intensity profiles, colocalization analysis, and morphological characterization, were performed on the unmixed images. Colocalization analysis was conducted using ZEN 3.3 software. Specific channels corresponding to different labels were selected for analysis utilizing the software's colocalization module. Thresholds were set to differentiate the signal from background noise, ensuring precise colocalization measurements. The degree of colocalization between the labeled structures was quantified using the colocalization coefficient known as Manders' coefficient. Subsequently, co-localization channels and scatterplots were generated to visualize and analyze the colocalization patterns.

### Immunoprecipitation

Stable cell lines expressing GFP and GFP-LC3A protein were seeded in 15-cm cell culture plates. After 48 h of LC3A expression induction with tetracycline, cells were washed with ice-cold PBS and lysed in I.P. lysis buffer (90409, Thermo Scientific). One milligram of total protein was combined with 10 μg of anti-GFP antibody (Abcam, ab6556) in Protein LoBind tubes (Eppendorf, 022431081) and incubated overnight at 4 °C while rotating. Next, the sample-antibody mixture was added to 0.25 mg of Pierce protein A/G Magnetic Beads to perform manual immunoprecipitation according to the manufacturer's instructions. Immunoprecipitated samples were eluted, dried in a speed vacuum concentrator, and reconstituted in urea sample buffer. The isolated proteins were resolved by 12% SDS-PAGE.

### Real-time analysis of cytotoxicity

Twenty-four hours before tetracycline-induction, 50 μl of complete medium was added to the electronic microtiter plate (E96) xCELLigence plate for impedance background measurement. Following harvesting and counting, stable clone cells were diluted to 10,000 cells/well and added to the 50 μl medium. The E-Plate was incubated at 37 °C with 5% CO_2_ and monitored with the RTCA software (xCELLigence Real-Time Cell Analysis) at 5-min intervals. The following day, the cell index was assessed to ensure an equivalent number of cells across all conditions. Subsequently, cells were treated with 1 μg/ml tetracycline, and the cell index was monitored for up to 100 h post-induction. CLQ was added to the cells at a final concentration of 50 μM, concurrently with tetracycline, to evaluate autophagy flux.

### SILAC and mass spectrometry (LC-MS/MS)

Stable isotope labeling of amino acids in cell culture (SILAC) was performed to quantitatively analyze the effect of LC3A expression on CCHE-45 cells. Control-CCHE-45 cells were labeled with “heavy” amino acid: 0.248 mg/ml L-13C6 Arginine-HCL (Cambridge Isotope Laboratories Inc, CLM-2265) and 0.04 mg/ml L-lysine-2HCL (Thermo Scientific, 88429). While the myc-LC3A and myc stable clones were labeled with the “light” amino acid L-arginine and L-lysine using 0.2 mg/ml and 0.04 mg/ml L-arginine, free base (Millipore Sigma,1820-100GM) and L-lysine-2HCL, respectively. SILAC-heavy and light-labeled cells were lysed and combined in a 1:1 ratio. Protein samples were reduced with 10 mM dithiothreitol in 50 mM ammonium bicarbonate for 30 min at 60 °C, alkylated in the dark with 55 mM iodoacetamide in 50 mM ammonium bicarbonate for 30 min at room temperature, and digested overnight at 37 °C with trypsin. The protein digestion reaction was stopped by acidification. Cells were collected from three biological triplicates for two time points (48 h and 96 h following expression induction with tetracycline). For LC/MS/MS, digested samples were analyzed using an EASY-nanoLC 1200 system and an Orbitrap Fusion Lumos Tribrid mass spectrometer (Thermo Scientific) coupled with a NanoFlex ion source. 100 ng of peptides were trapped on Acclaim PepMap 100 C18 HPLC trap columns (Thermo Scientific, 164750) with 75 μm I.D., 2 cm length, and 3 μm particles. Then, peptides were eluted on an Acclaim PepMap 100 analytical column (Thermo Scientific, 164569) with a 75 μm I.D., 25 cm length, 2 μm particles, and C18 packing. Sample elution was performed using the one-hour gradient of solvent B (0.1% formic acid, 80% acetonitrile) that was conducted at 5% to 30% for (0–31 min), 30% to 40% for (31–41 min), 40% to 80% for (41–51 min), held for 4 min at a flow rate of 250 nl/min, and followed by a 5-min ramp to 100%. Then, solvent A contained 0.1% formic acid in water. The mass spectrometer was operated in data-dependent acquisition (DDA) mode with 3-s cycles for the survey and the MS/MS scans. Survey scans of peptide precursors were performed from 400 to 1800 m/z at 120K resolution with standard automatic gain control (AGC) and maximum ion injection time (I.T.) set to auto mode. Monoisotopic precursor selection (MIPS) was determined at the peptide level with an intensity threshold of 5 × 103, and only peptides with charge states of 2 to 7 were selected for tandem M.S. The dynamic exclusion was set to 30 s with a 10 ppm mass tolerance, and isotopes were excluded. Isolation for MS2 scans was performed in the quadrupole with an isolation window of 1.5 m/z. Higher-energy collisional dissociation (HCD) activation was applied with 30% collision energy using dynamic injection time mode and a standard AGC target. The resulting fragments were detected using the rapid scan rate in the linear ion trap. The MS1 and MS2 spectra were recorded in profile and centroid modes. The mass spectrometry proteomics data have been deposited to the ProteomeXchange Consortium *via* the PRIDE [1] partner repository with the dataset identifier PXD043703.

### Proteomics data analysis, gene enrichment, and subcellular analysis

MaxQuant (version 1.6.17) was used to identify and quantify peptides, matching against the reference human proteome (uniprot_cano_varsplic_HUMAN) supplemented with the default contaminants database. Relevant settings for analysis included: the use of the heavy label feature (set to Arg6), variable modification of methionine oxidation and acetylation of the protein N terminus, along with the use of trypsin specificity with a maximum of two missed cleavages, FDR limited to 1% at both the peptide and protein levels, and unique + razor peptides selected for quantification while all other settings were set to the default. For differential abundance analysis, the resulting evidence.txt and proteingroups.txt files were analyzed using aggregation, normalization, and the differential expression analysis tools in ProteoSign5 (version 2.0) using default parameters. After removing the contaminants, DEPs were identified using logFC (1.5 and −1.5), and the change expression between biological replicates was tested (at adjusted *p* ≤ 0.05). Pearson’s correlation scores were calculated between the different biological replicates in the proteomics experiments. The correlation scores were calculated based on the abundance values of identified proteins in each sample. A high correlation score indicates a strong agreement between the replicates, whereas a low score indicates a significant variation between them. These scores are useful in assessing the reproducibility and reliability of proteomics experiments and can guide the selection of appropriate replicates for downstream analysis. The circular clustered heatmap was plotted using the SRplot tool https://www.bioinformatics.com.cn/en. The enrichGo6 function in the (cluster Profiler) R package was used to perform functional enrichment analysis of DEPs. Gene ontology (GO) in three sections: biological process (BP), molecular function (MF), and cellular component (CC), were identified based on (Benjamini-Hochberg FDR <0.05) with the removal of redundant terms. The ClueGO7 plugin in Cytoscape software was employed to visualize the significant GO terms. Subcellular analysis was performed using SubcellularRVis, a bioinformatics tool that describes subcellular localization for a gene list (46). SubcellulaRVis can be accessed *via* the web (http://phenome.manchester.ac.uk/subcellular/).

### Senescence-associated-ß-galactosidase (SA-ß-Gal) staining assay

ß-Gal activity was determined using the SA-β-Gal Staining Kit (Invitrogen, K1465-01). Both the CCHE-45 and HEK293 cell lines were subjected to different conditions: control, transfected with the pcDNA3.1/His/LacZ vector, tetracycline-treated cells, and stable clones. After 24 h of seeding cells in 6-well culture plates, tetracycline was added for 1, 2, 3, 4, 7, and 14 days.

### Statistical analysis

GraphPad Prism version 8 software was used for statistical analysis. Image analysis was performed using Fuji. Error bars were plotted as standard errors of the mean (±SD) for three independent biological experiments. A two-tailed Student's *t* test was performed for real-time PCR, immunoblot quantification, and comparisons. One-way ANOVA was used to compare the means between control and GFP-LC3A-induced cells.

## Data availability

All data generated during this study are included in this article and its supporting information. The mass spectrometry proteomics data have been deposited to the ProteomeXchange Consortium *via* the PRIDE [1] partner repository with the dataset identifier PXD043703.

## Supporting information

This article contains supporting information.

## Conflict of interest

The authors declare that they have no conflicts of interest with the contents of this article.

## Supporting information

Movie 3

Supplemental Figures S1–S4, Tables S5 and S6, and Tables S1–S4 Caption
