# Supplementary material for: LC3A-mediated autophagy elicits PERK-eIF2α-ATF4 axis activation and mitochondrial dysfunction: Exposing vulnerability in aggresome-positive cancer cells
Source: J Biol Chem. 2024 May 20;300(6):107398. doi: 10.1016/j.jbc.2024.107398 (PMC11227016; doi:10.1016/j.jbc.2024.107398)
Supplement: Supplemental Figures S1–S4, Tables S5 and S6, and Tables S1–S4 Caption [file mmc4.docx]

**Supplementary Information**

**LC3A-mediated autophagy elicits PERK-eIF2α-ATF4 axis activation and mitochondrial dysfunction: Exposing vulnerability in aggresome positive cancer cells**

**Authors**

Nada Amer ^1,2,^ Dina Hesham ^1,2,^ Nouran Al-Shehaby ^1^, Hisham A. Elshoky ^1^, May Amer^1^, Sameh Magdeldin ^3,4^, Manar Mansour ^2^, Khaled Abou-Aisha ^2^ and Shahenda El-Naggar ^1*^

1. Tumor Biology Research Program, Basic Research Unit, Research Department, Children’s Cancer Hospital Egypt 57357, Cairo, Egypt.
2. Faculty of Pharmacy and Biotechnology, German University in Cairo (GUC), Egypt.
3. Proteomics and Metabolomics Research Program, Basic Research Unit, Research Department, Children’s Cancer Hospital Egypt 57357, Cairo, Egypt.
4. Department of Physiology, Faculty of Veterinary Medicine, Suez Canal University, Ismailia, Egypt.

* **Corresponding Author**: Dr. Shahenda El-Naggar, Tumor Biology Research Program, Basic Research Unit, Research Department, Children’s Cancer Hospital in Egypt 57357, 1 Sekket El Emam, El Madbah El Kadeem Yard, Sayeda Zeinab, Cairo, Egypt.
E-mail: [shahenda.elnaggar@57357.org](mailto:shahenda.elnaggar@57357.org)

Phone: +20 25351500

ORCID ID: 0000-0001-6465-9881.

**Keywords**

Autophagy; Endoplasmic reticulum; Inclusion bodies; MAP1LC3A; Protein quality control; Proteomics; Proteostasis; Senescence.

**Supplementary Figures:**

Supplementary Fig.S1:

**Supplementary Fig.S1:**

**(A)** Cloning vector maps showing control vector “pcDNA4/TO/His A” and cloned myc*LC3Av*1, GFP, and GPF-*LC3Av1*. **(B)** mRNA expression levels of myc-LC3A (red) and GFP-LC3A (green) after LC3A induction with tetracycline in CCHE-45 and HEK293 cells. Data are presented as mean ± SD. ***p ＜0.001 **(C)** Double immunofluorescence staining for LC3A (in red) and vimentin (in green). The specificity of LC3A antibodies was assessed in myc-LC3A cells without tetracycline induction. Scale bar is 10 µm. (**D**) A three-dimensional Z-stack image of GFP-LC3A-induced CCHE-45 cells, highlighting LC3A positive puncta (on the left panel, with scale bar is 5 µm). On the right panel, an immunofluorescence colocalization analysis for lysosomes using LysoTracker (Red) along with GFP-LC3A puncta, with scale bar is 10 µm. Linear unmixing and co-localization analysis were performed using ZEN blue software. **(E)** HEK293 cells expressing myc-LC3A were fixed and immunolabeled with anti-LC3A (red). For GFP-LC3A expressing cells, the cells were fixed and visualized for GFP signal with no further immunostaining. Scale bar is 10 µm (**F**) Immunofluorescence staining in HEK293 cells for LC3A (green) and vimentin (red) in control cells (scale bar is 5 µm) is and cells expressing GFP-LC3A under normal conditions (scale bar is 10 µm) and following MG132 treatment (scale bar is 5 µm). (**G**) Immunofluorescence staining for LC3A (red) and LC3B (green) in SH-SY5Y control cells, after serum starvation (S.S) for 5 hours, and serum starvation with the addition of chloroquine (CLQ) for 5 hours. Scale bar is 10 µm. (**H**) Immunofluorescence staining for LC3A (green) and vimentin (red) in SH-SY5Y control cells (scale bar is 10 µm) and after the MG132 treatment (scale bar is 5 µm). In all immunofluorescence staining, DAPI was used for nuclear staining (**I**) Impedance monitoring was conducted for the following conditions: CCHE-45 cells stably transfected with GFP, GFP-LC3A, myc, and myc-LC3A. The X-axis represents time in hours, with cell monitoring conducted for up to 100 hours. The Y-axis represents the cell index obtained from the RTCA software. Three biological replicates were performed and the data is presented as mean ± SD.

Supplementary Fig.S2:

**Supplementary Fig. S2:**

(**A,** **B**) Pearson’s correlation scores for the SILAC biological replicates following myc-LC3A induction for 48 and 96 hours, respectively. **(C)** Venn diagram for the number of significant, unique, and shared proteins identified at 48- and 96-hours following induction of LC3A expression. (**D**) Heatmap representation of DEPs after induction of LC3A for 48 and 96 hours. The heatmap illustrates the relative expression levels of the DEPs as the log2 of the light/heavy ratio. The color gradient indicates the expression levels, with green representing downregulated DEPs and red indicating upregulated DEPs. **(E)** Gene Ontology of exclusive DEPs after 48 hours of LC3A expression highlights the enriched biological processes, molecular functions, and cellular components specific to this time point. **(F)** Gene Ontology of exclusive DEPs after 96 hours of LC3A expression, highlighting enriched biological processes, molecular functions, and cellular components.

Supplementary Fig.S3:

**Supplementary Fig. S3:**

Scanned Western blots for the UPR detection in CCHE-45.

Supplementary Fig.S4:

**Supplementary Fig. S4:**

Scanned Western blots for the UPR detection in HEK293.

**Supplementary Tables:**

**Table S1:** Table summarizing counts and size for GFP-LC3A. Counts for myc-LC3A positive cells and their localization with aggresomes, Co-localization analysis of GFP-LC3A with lysosomes along with the flow cytometric data of the CellTrace violet.

**Table S2:** Proteomics SILAC data of myc-LC3A for the 48 and 96 hours’ time point along with myc-tag stable clone for the 48 and 96 hours’ time. The table includes protein identification information, the abundance of each identified protein in each sample, and associated statistical measures.

**Table S3:** Subcellular localization analysis of the DEPs of the myc-LC3A after 48 and 96 hours of the induction using SubcellularRVis. The table includes the subcellular compartments predicted by the tool and the corresponding number of DEPs predicted to localize to each compartment. The subcellular compartments are ordered based on their p-value, with the most significantly enriched compartment listed first.

**Table S4:** Gene Ontology of DEPs exclusive after 48 and after 96 hours of LC3A expression along with the common DEPs between two time points. The table lists the specific biological processes, molecular functions, and cellular components that are enriched among these DEPs with their adjusted p-values, and the IDs of the involved genes.

**Table S5:** List of oligonucleotide primers.

| **Primer Name** | **Sequence** |
| --- | --- |
| LC3AV1- Forward | 5′- CCTCAGACCGGCCTTTCAA -3′ |
| LC3AV1- Reverse | 5′- AGCTGCTTCTCACCCTTGTA -3′ |
| Beta-Actin- Forward | 5′- CTGAAGTACCCCATCGAGCA -3′ |
| Beta-Actin- Reverse | 5′- AGCCTGGATAGCAACGTACA -3′ |
| BiP (GRP-78) - Forward | 5′- GGAAAGAAGGTTACCCATGC -3′ |
| BiP (GRP-78) - Reverse | 5′- TTAGGCCAGCAATAGTTCCA -3′ |
| ATF4 - Forward | 5′- CCAAGCACTTCAAACCTCATG -3′ |
| ATF4 - Reverse | 5′- ATCCATTTTCTCCAACATCCAATC -3′ |
| Chop - Forward | 5′- GTACCTATGTTTCACCTCCTGG -3′ |
| Chop - Reverse | 5′- TGGAATCTGGAGAGTGAGGG -3′ |
| XBP1 - Forward | 5′- TGGCCTTGTAGTTGAGAACCA -3′ |
| XBP1 - Reverse | 5′- ATGGGGAGATGTTCTGGAGG -3′ |

**Table S6:** List of reagents and antibodies.

| **Name** | **Catalog Number** | **Dilutions/ Concentration** |
| --- | --- | --- |
| Rabbit anti- LC3A (D50G8) | (4599, Cell Signaling) | 1:100 (IF)/ 1:1000 (WB) |
| Rabbit anti-MAP1LC3A [EP1983Y] | (ab52768, Abcam) | 1:100 (IF) |
| Mouse anti-MAP1LC3A [BS405] | (ab244210, Abcam) | 0.5 µg (IF) |
| Rabbit Cleaved LC3A Antibody | (AP1805a, Abcepta) | 1:50 (IF) |
| Rabbit-LC3B (D11) | (3868, Cell Signaling) | 1:100 (IF) |
| Mouse Anti-LC3B [GT3612] | (ab243506, Abcam) | 1:200 (IF) |
| Rabbit anti-GFP | (ab6556, Abcam) | 10 µg (IP) |
| Rabbit anti-GFP (D5.1) | (2956, Cell Signaling) | 1:1000 (WB) |
| Mouse anti-Myc tag [Myc.A7] | (ab18185, Abcam) | 1:1000 (WB) |
| Mouse anti-GRP 78 (E-4) | (sc-166490, Santa Cruz) | 1:1000 (WB) |
| Mouse anti-PERK (B-5) | (sc-377400, Santa Cruz) | 1:1000 (WB) |
| Rabbit anti-p-PERK (Thr 981) | (sc-32577, Santa Cruz) | 1:500 (WB) |
| Mouse anti-eIF2α (D-3) | (sc-133132, Santa Cruz) | 1:1000 (WB) |
| Rabbit anti-Phospho-eIF2a (Ser51) | (701268, Invitrogen) | 1:500 (WB) |
| Rabbit anti-ATF-4 (D4B8) | (11815, Cell Signaling) | 1:1000 (WB) |
| Rabbit anti-IRE1 alpha Antibody | (NB100-2324, Novus Bio) | 1:1000 (WB) |
| Rabbit anti-IRE1 alpha [p Ser724] | (NB100-2323, Novus Bio) | 1:500 (WB) |
| Mouse Anti-ATF6 [1-7] | (ab122897, Abcam) | 1:1000 (WB) |
| Mouse Anti-Vimentin [RV200] | (ab8978, Abcam) | 1:100 (IF) / 1:1000 (WB) |
| Rabbit Anti-Vimentin (D21H3) | (5741S, Cell Signaling) | 1:50 (IF)/ 1:1000 (WB) |
| Rabbit Anti-Cytokeratin 8 [EP1628Y] | (ab53280, Abcam) | 1:1000 (WB) |
